# Revealing the Mechanical Properties and Fracture Mechanism of Ag Paste Sintered Solder by Two Different Preparation Methods

**DOI:** 10.3390/ma18071435

**Published:** 2025-03-24

**Authors:** Jialong Liang, Hao-Kun Yang, Xingming Huang, Li-Yin Gao, Zhi-Quan Liu

**Affiliations:** 1 Shenzhen Institutes of Advanced Technology, Chinese Academy of Sciences, Shenzhen 518055, China; 2 University of Chinese Academy of Sciences, Beijing 100049, China; 3 Smart Manufacturing Division, Hong Kong Productivity Council, Hong Kong SAR, China

**Keywords:** semi-sintered Ag paste, sintered Ag-Cu joint, in situ tensile and shear tests, fracture mode, crack

## Abstract

This paper studied the microstructure and mechanical properties of sintered Ag-Cu joints and semi-sintered Ag-Cu joints under tensile and shearing actions. By a comparative analysis of the differences in sintered Ag layer microstructure characteristics, crack propagation directions, and fracture surface characteristics, the differences in the fracture mechanisms of the two types of sintered joints under the influence of tensile and shearing forces were further explained. Research shows that the sintered Ag joints have higher tensile and shearing properties than the semi-sintered Ag joints, but the two show similar fracture modes under the action of tensile force. The same phenomenon also occurs in the fracture mechanism under the action of shearing force. The difference is that under the action of tensile stress, cracks in the joints initiate from the Ag-Cu interface and grow along the interface until fracture occurs, while under the action of shearing force, cracks in the joints still initiate from the Ag-Cu interface, but then turn, and the cracks grow along the silver layer towards another Ag-Cu interface, which is a composite fracture mode. Under the action of tensile stress, the adhesive force of the Ag-Cu interface is shown to be weaker than the cohesive force within the sintered Ag layer itself, and the sintered Ag layer shows better ductile deformation. Under the action of shearing force, the advantage of the cohesive force within the sintered Ag layer is weakened, and the sintered Ag layer begins to fracture. Since sintered Ag joints have a better Ag-Cu interface and lower porosity than semi-sintered Ag joints, the energy required for failure increases correspondingly, showing better mechanical properties.

## 1. Introduction

Within recent years, high-density integration continuously develops in the electronics industry, resulting in heat being generated from chips. Thus, the design of printed circuit boards (PCBs) should be more resistant to high temperatures [1]. Nowadays, Ag sintered solder is one of the promising porous interconnection materials with advantages of (1) low-temperature sintering requirement, (2) high melting point of solders, and (3) excellent electrical and thermal conductivity [2,3]. Nowadays, the sintered Ag bonding quality on the surface of Cu substrate is becoming more and more important for the resistance of heat damage in PCB design. This is because the interface between Ag and Cu substrates easily forms different intermetallic compound (IMC) layers during the long operation time [4]. In addition, these substrates (composed of different elements) have various coefficients of thermal expansion. Thus, the internal stress, induced by the fabrication and operation process, will cause non-negligible damage to PCBs. It thus appears that the re-design of Ag sintered solder should be one of the challenges to consider.

To further investigate the effect of processing factors on the Ag sintered solder’s strength, Bai and Lu [5] found that the shear strength and structural densities of sintered Ag joints increased when the sintering time extended beyond 40 min, as the sintering temperature affects the volatilization of organic matter within the Ag paste [6]. Mohd Zubir et al. [7] obtained good connection quality when using organic-free Ag paste for large-area chip interconnection. Stuckner et al. [8] suggested that, at higher sintering temperatures, the structure of a sintered body would become denser. Liu et al. [9] found that the greater sintering pressure, the lower the porosity of the sintered Ag and the higher shear quality. Zhao et al. [4] elucidated that the higher interface connection ratio, the less the contact angle of the Ag sintered solder can further improve the crack propagation resistance. Xu et al. [1] pointed out that the Ni/Au substrate further strengthens the die-attachment. Fan et al. [10] believe that the inter-diffusion rate between gold atoms and silver atoms is higher than the self-diffusion rate of silver atoms, resulting in the formation of pores at the interface. Zhang et al. [2] investigated the methods of preheating treatment on substrate and enhancing the subtract printing thickness for bonding strength improvement. The addition of resin has been reported to alleviate the problem of reduced joint strength due to the Ostwald ripening mechanism [11].

At present, the fully sintered process, with a sintering temperature of 250 °C to 300 °C, has been relatively mature. However, its cost and process requirements are relatively high, so it is mostly applied in high-temperature and high-power devices such as automotive electronics, and it does not particularly have a place in the application of small- and medium-power devices such as sensors and flexible electronic devices. Therefore, some scholars have proposed adding organic resin to the silver paste. By using the mechanical fastening effect of resin curing, that is, the cured resin adheres to the sintering necks or fills the pores in the sintered silver, the bonding strength of the sintered silver interconnection is enhanced, ensuring the high-strength connection of the silver sintered joints at a lower sintering temperature (150–250 °C). At the same time, the silver powder content in the silver paste can be reduced by 20%, which reduces the cost. de Wit [12] labeled this polymer-assisted semi-sintered silver sintering method as semi-sintering and looked forward to its application prospects in the automotive electronics industry.

In recent years, there has been a number of studies on sintered silver with the addition of resins. Jung et al. [13] studied the influence of adding different proportions of epoxy resin to nano silver solder paste on the properties of sintered joints. When the mass ratio of epoxy resin increased from 4.5% to 18%, the shear strength of the joint increased from 5.18 MPa to 45.59 MPa, showing an improvement in mechanical strength. Sasaki and Mizumura [14] found that by adding epoxy resin to nano silver solder paste, a silver sintered joint with a sintering temperature of 200 °C can be achieved, and its shear strength is as high as 30 MPa. Subsequently, Sasaki and Mizumura [15] added a thermoplastic resin to Ag paste and sintered it on bare copper to obtain a good bond strength. Jin et al. [16] achieved bonding under no-pressure conditions by adjusting the ratio of micron-sized silver flakes to thermosetting resin, and the shear strength on the bare silicon wafer was equivalent to that on the gold-plated silicon wafer. The resin-enhanced method can achieve sintering at lower temperatures and improve the bonding strength of the interconnection, but it also has obvious limitations. Due to the large difference in the coefficient of thermal expansion between the resin and the sintered silver, cracks will be generated in a high-temperature environment, and the resin will decompose in a high-temperature environment, and its shear strength will also decrease accordingly. At the same time, since the resin exists as an organic matter in the sintered body, the electrical and thermal conductivity of the joint will also be reduced. Liang et al. [17] found that during aging at 200 °C, the coarsened Ag squeezes the resin, resulting in the failure of the bonding interface. Jung et al. [13] discovered that the higher the amount of epoxy resin in the silver paste, the lower the electrical conductivity of the sintered joints. In summary, at present, there has been a certain amount of research on the proportion, sintering behavior, reliability and electrical conductivity of both Ag paste and semi-sintered Ag paste. However, there is still a lack of specific research on the differences in the fracture behavior of these two types of Ag paste. In order to further reveal the potential fracture mechanism of semi-sintered Ag sintered solder, in situ tensile and shear tests were carried out, and the fracture surfaces were analyzed.

## 2. Materials and Methods

### 2.1. Sample Preparation

**Figure 1:**
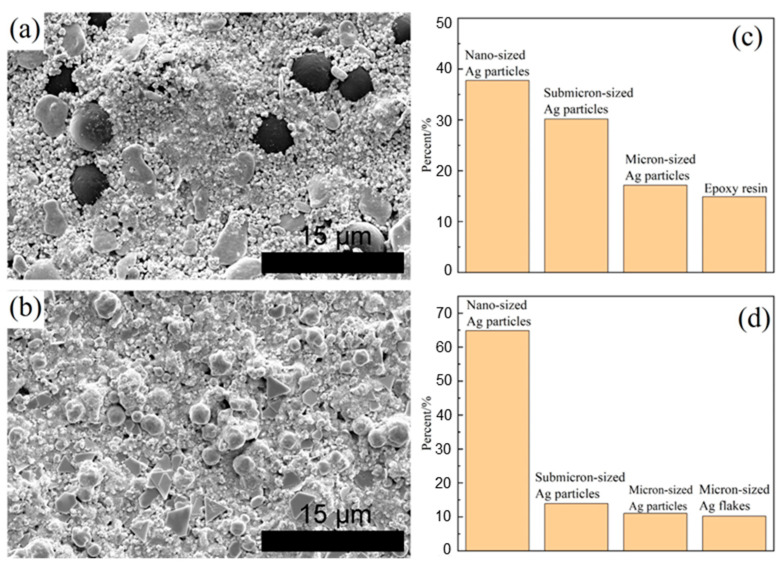
Microstructures and particle proportion of (**a**,**c**) semi-sintered Ag paste and (**b**,**d**) Ag paste.

The semi-sintered Ag paste used in this article was sourced from TANAKA PRECIOUS METAL Company in Tokyo, Japan, and the Ag paste was from Nihon Handa Company in Funabashi, Japan. The micro components and structures of the silver paste were observed by scanning electron microscopy. More than ten photos were taken for each type of silver paste, and the particle size was counted using the software ImageJ (Version: ImageJ-150-win-java8). The results are shown in Figure 1.

Semi-sintered Ag paste is composed of micron-sized Ag particles (average diameter 2.4 ± 0.28 μm), submicron-sized Ag particles (average diameter 0.4 ± 0.047 μm), nanometer-sized Ag particles (average diameter 0.08 ± 0.0096 μm), and micron-sized resin (average diameter 4.8 ± 0.576 μm), which translates to a solder thickness of 1 mm.

Ag paste is composed of micron-sized thin Ag flakes (average diameter 1.67 ± 0.21 μm), micron-sized Ag particles (average diameter 1.85 ± 0.22 μm), submicron-sized Ag particles (average diameter 0.3 ± 0.036 μm), and nanometer-sized Ag particles (average diameter 0.05 ± 0.0051 μm), which translates to a solder thickness of 0.1 mm.

**Figure 2:**
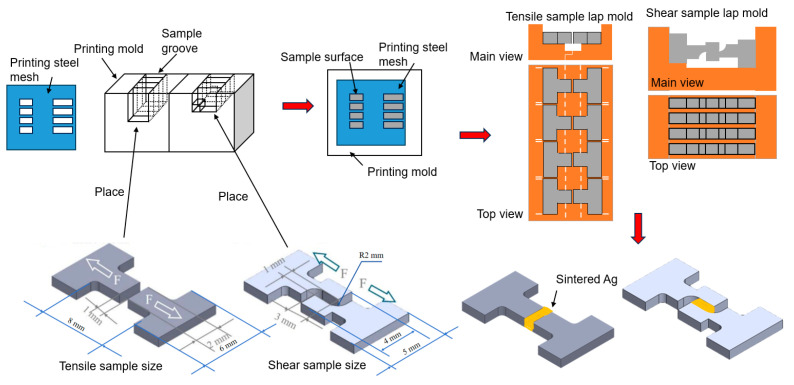
Sample preparation schematic.

The preparation process of the sintered samples is as follows:

(1) The in situ tensile/shear samples were machined from pure copper according to the designed dimensions by the electrical discharge machining (EDM) method, with the precision controlled within 10 μm. Subsequently, the samples were fixed on the fixture to ensure good contact between the workpiece and the electrodes. The workpiece was immersed in the electrolyte, and the power was turned on. Polishing was carried out at a voltage of 7 V for 1 min. Under the action of the electric field, the redox reaction of metal ions in the electrolyte occurs, and the microscopic protrusions on the metal surface are preferentially dissolved, thus achieving surface smoothing and removing oxides and contaminants on the sample surface. Immediately after polishing, the workpiece was taken out from the electrolyte and thoroughly washed with deionized water to remove the residual electrolyte.
(2) The process of printing silver paste is shown in Figure 2. The samples were placed and fixed in the corresponding grooves of the mold. The printing steel mesh was placed on the surface of the printing mold and aligned with the printing area of the fixture, and then the silver paste was printed on the sample surface. Four samples could be printed and prepared at once. The thicknesses of the steel mesh were controlled at 0.1 mm and 1 mm, respectively. The openings in the printing area need to match the contact surfaces of the shear and tensile mold, and the dimensions of the samples are as shown in Figure 2. Subsequently, the printed tensile samples and the non-printed samples were fixed in the left and right molds, respectively. There are positioning lines on both sides of the mold to prevent misalignment after fitting. There are grooves in the middle part of the mold to prevent the sintered silver paste from contacting the mold. The two molds are assembled together through the shape matching of their bases to complete the lapping. For the shear samples, the printed samples with the concave side up were placed in the left-side groove of the mold, and then the other samples with the concave side down were placed in the right side groove and pressed tightly to achieve lapping with the left-side samples.
(3) The lapped samples were placed in the furnace for sintering. The sintering temperature was 300 °C, the sintering time was 60 min, and the heating rate was 5 °C/min. The sintering process was carried out without pressure in N_2_ atmosphere. In the present study, pre-heating at 150 °C for 10 min was required to reduce the influence of the solvent on the silver paste.

### 2.2. In Situ Tensile and Shear Tests

**Figure 3:**
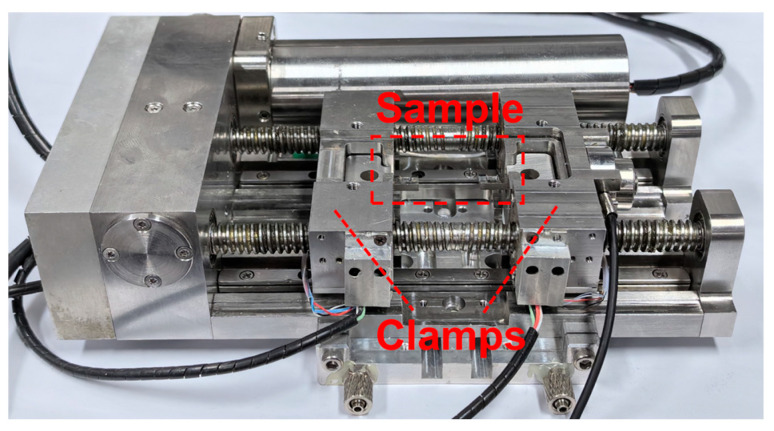
Tensile and shear test equipment.

The shear and tensile tests were commissioned to Changsha Kaiple New Material Company in Changsha, China for completion. The shear and tensile test bench equipment are shown in Figure 3. Two tensile tests and two shear tests for each type of sintered Ag joints. Before the start of the tests, first, by adjusting the width of the clamps, the positions of the left and right clamps were made suitable for clamping samples of different sizes. Then, both ends of the sample were clamped between the left and right clamps. During the experiment, the drive shaft was driven by the motor to apply a load to the samples, and the absolute position code was read by the optical encoder on one side of the drive shaft. The load sensor collected the test data and recorded it in the computer. The shear and tensile tests were all carried out at room temperature, and loading speed was 2 μm/s. The equipment used to observe silver paste particles in this article was Thermo Fisher Scientific, Apreo 2, Waltham, MA, USA. The in situ shear and tensile tests were carried out in the scanning electron microscope (CIQTEK, SEM5000, Hefei, China), which was equipped with EDS (Oxford Instruments Ultim Max, Oxford, UK). The model of the shear and tensile test bench is PicoFemto—SEP, SEM5000 N, Anhui, China. The model of the optical microscope is Olympus, BX51, Hachioji, Japan. The thermogravimetric analyzer is METTLER TOLEDO, Thermal Analysis System 2, Greifensee, Switzerland.

## 3. Results and Discussion

### 3.1. Original Microstructure of Ag-Cu Sintering Joints

**Figure 4:**
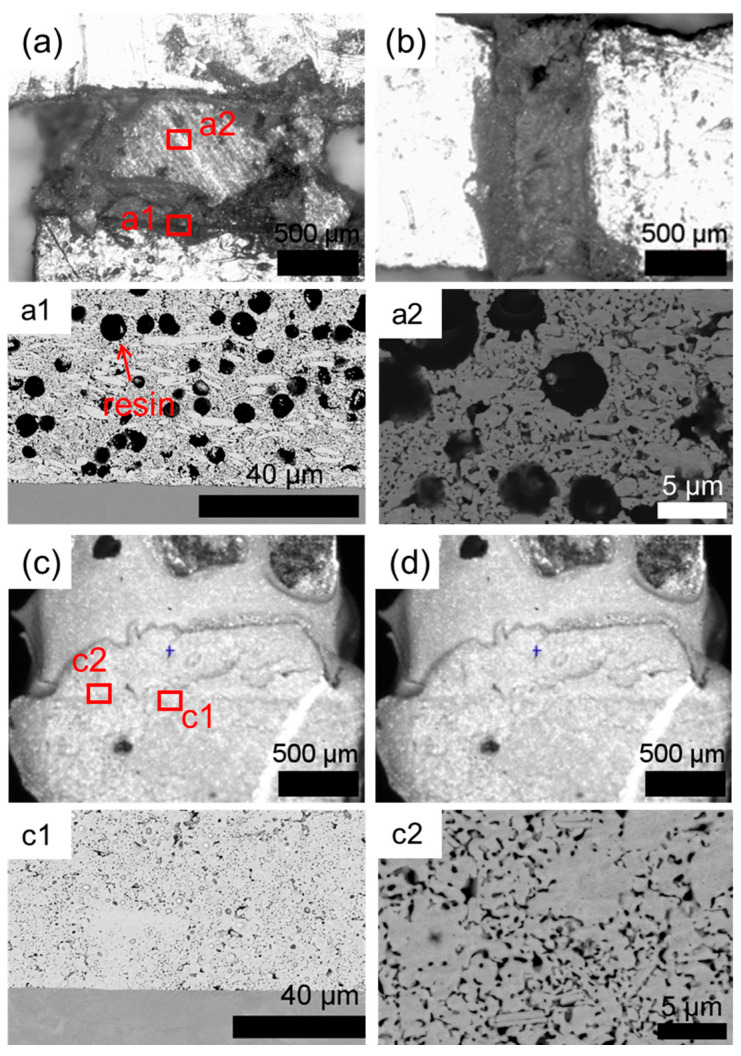
The optical microscope and SEM micromorphologies of the semi-sintered Ag joints: (**a**) tensile test, (**b**) shear test; and sintered Ag joints: (**c**) tensile test, (**d**) shear test. (**a1**,**a2**) and (**c1**,**c2**) are magnified views of local areas in (**a**) and (**c**), respectively.

The optical microscope and SEM micromorphologies of the Ag sintered joints and semi-sintered Ag joints are shown in Figure 4. It can be seen from Figure 4a,b that in the semi-sintered Ag joints of the tensile and shear samples, a good connection is formed between the sintered Ag layer and the copper substrate, and there are no obvious cracks. The same morphology can be observed in the Ag sintered silver joints in Figure 4c,d. In Figure 4(a1,a2) are the Ag-Cu interface microstructure morphology and the sintered Ag body micro morphology of the semi-sintered Ag joint, respectively. It can be found that there are a lot of unvolatilized resins in the semi-sintered Ag structure, and many particles still retain their original particle shapes, such as spherical particles and flake-like particles, and a certain connection is generated between the sintered Ag layer and the Cu substrate. It can be observed in Figure 4(c1,c2) that the sintered Ag structure has a sintered silver layer with a uniform, microporous, continuous block-like structure and a higher density.

### 3.2. In Situ Tensile and Shear Testing

**Figure 5:**
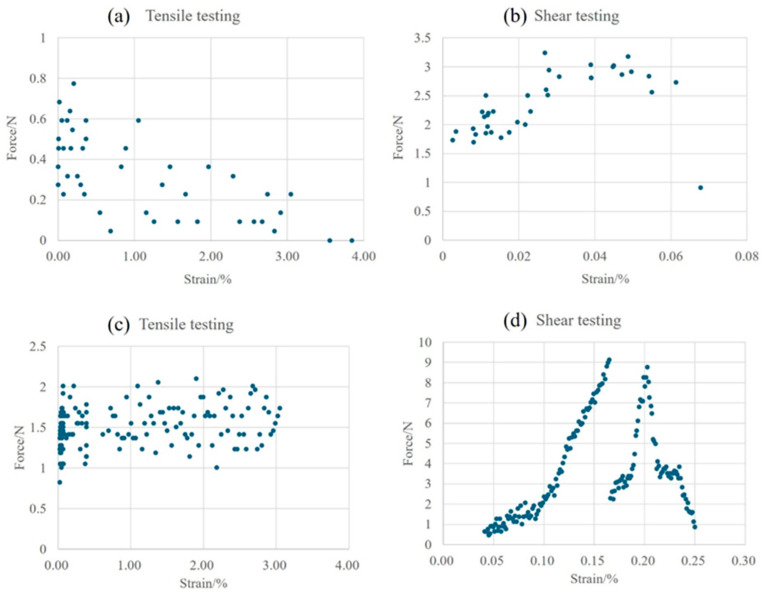
The in situ tensile and shear testing of semi-sintered Ag joints: (**a**) tensile test, (**b**) shear test; and sintered Ag joints: (**c**) tensile test, (**d**) shear test.

**Figure 6:**
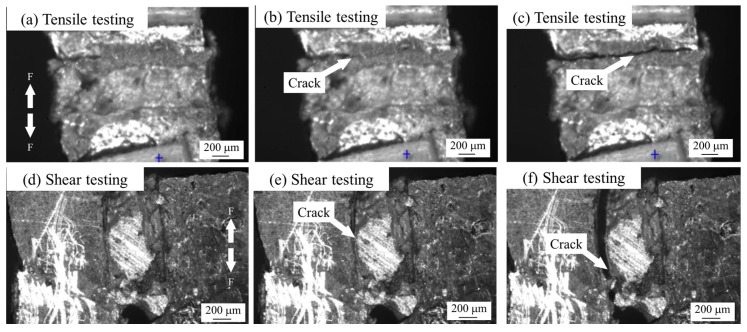
The in situ tensile and shear testing observations of semi-sintered Ag joints: (**a**–**c**) tensile testing, and (**d**–**f**) shear testing.

The tensile and shear test curves are shown in Figure 5. The tensile and shear strengths of the sintered Ag joints are higher than those of the semi-sintered Ag joints. As shown in Figure 6a,c respectively, the tensile strengths of the two types of sintered Ag joints show a trend of decreasing and then stabilizing with the strain. In Figure 6b,d, the shear curves of the semi-sintered Ag joints first rise and then fall with the strain, while the sintered Ag joints decline after an interruption in the middle of the curve. This potential mechanical property needs further observation and analysis.

**Figure 7:**
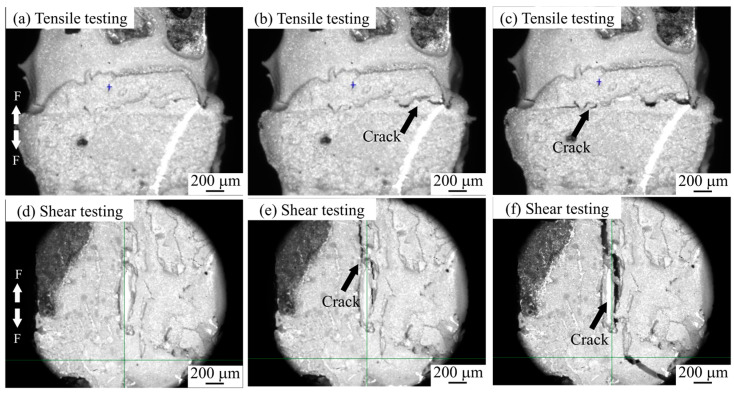
The in situ tensile and shear testing observations of sintered Ag joints: (**a**–**c**) tensile testing, and (**d**–**f**) shear testing.

The processes of shear and tensile tests of semi-sintered Ag joints and sintered Ag joints under an optical microscope are shown in Figure 6 and Figure 7. During the tensile process of the semi-sintered Ag joints in Figure 6a–c, cracks first occur at the interface between the sintered Ag body and the Cu substrate, and then the cracks propagate until the entire Ag-Cu interface fractures. During the shear process of the semi-sintered Ag joints in Figure 6d–f, cracks also first occur at the Ag-Cu interface and then propagate until fracture. The same Ag-Cu interface fracture mode can be observed during the tensile process of the sintered Ag joints in Figure 7a–c. The difference is that during the shear process of the sintered Ag joints in Figure 7d–f, cracks first occur at the Ag-Cu interface and then deflect towards the interior of the sintered Ag body. This is the reason for the interruption in the shear curve. Previous studies have shown that crack deflection can absorb external damage and improve the toughness of the overall fracture [18].

**Figure 8:**
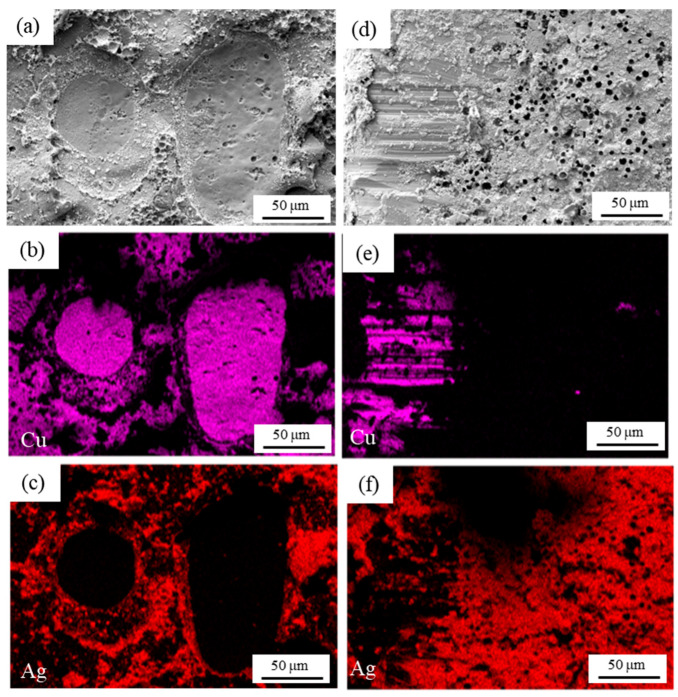
Scanning electron microscope observations of solder fracture of (**a**–**c**) tensile and (**d**–**f**) shear testing with semi-sintered Ag joints.

**Figure 9:**
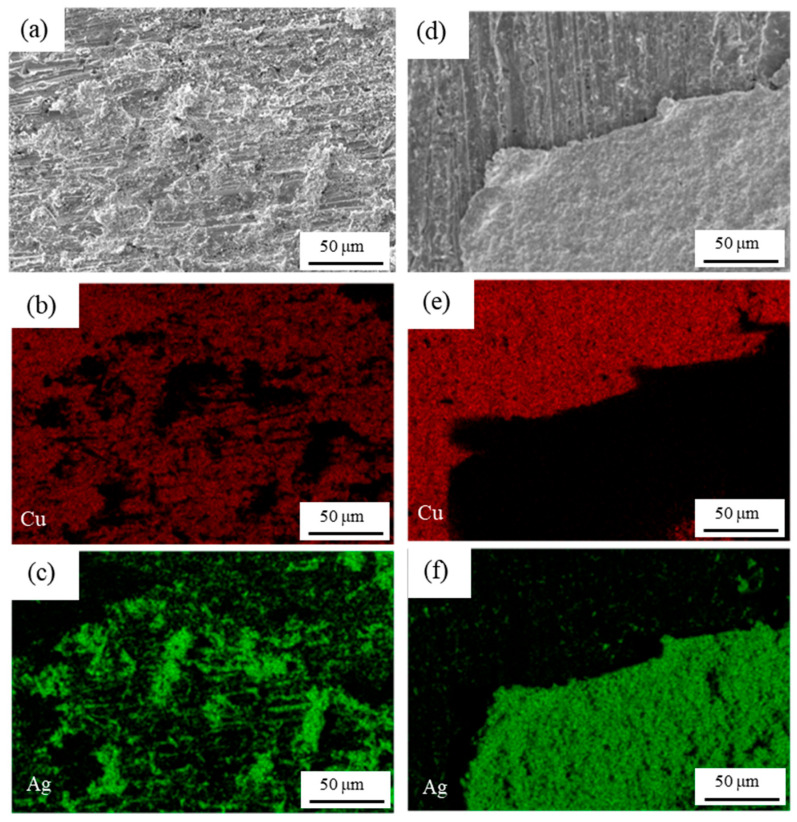
Scanning electron microscope observations of solder fracture of (**a**–**c**) tensile and (**d**–**f**) shear testing with sintered Ag joints.

To understanding of the fracture behavior, it is necessary to observe tensile fractures and shear fractures. As shown in Figure 8 and Figure 9, SEM observation of the fracture surfaces of semi-sintered Ag joints and sintered Ag joints, and the distributions of Ag and Cu elements are given, respectively. In the tensile fracture surface of the semi-sintered Ag joint in Figure 8a–c, a large area of the Cu substrate is exposed, and Ag-Cu interface fracture has occurred. In the shear fracture surface of the semi-sintered Ag joint in Figure 8d–f, both the sintered Ag layer and the copper substrate exist, and holes left by unvolatilized resins can still be seen in the remaining sintered silver layer. Thus, it is a composite fracture mode, that is, both sintered Ag layer fracture and Ag-Cu interface fracture have occurred. The same Ag-Cu interface fracture morphology can be observed in the tensile fracture surface of the sintered Ag joints in Figure 9a–c, but the exposed Cu substrate area of the semi-sintered Ag joint is larger, indicating that the connection between the sintered Ag and the substrate is better than that of the semi-sintered Ag joints. The shear fracture morphology in the sintered Ag joint in Figure 9d–f is also a composite fracture mode. In summary, the fracture modes of both sintered Ag joints in tensile tests are Ag-Cu interface fractures, and the fracture modes in shear tests are composite fractures. The influence of their microstructures on the fracture mechanisms of the two sintered Ag joints needs to be further discussed and analyzed.

### 3.3. Fracture Mechanism Analysis

**Figure 10:**
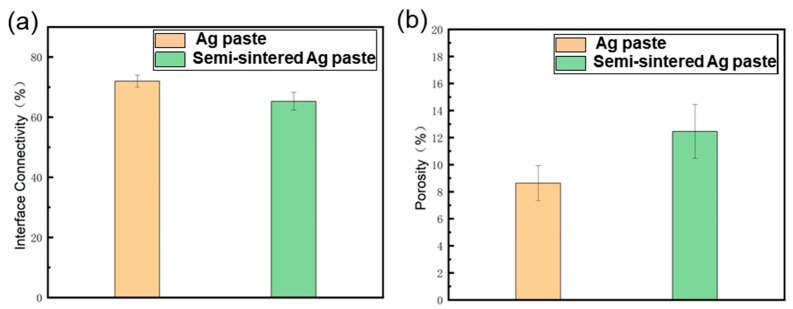
Two types of sintered joints: (**a**) Interface Connectivity; (**b**) Porosity.

**Figure 11:**
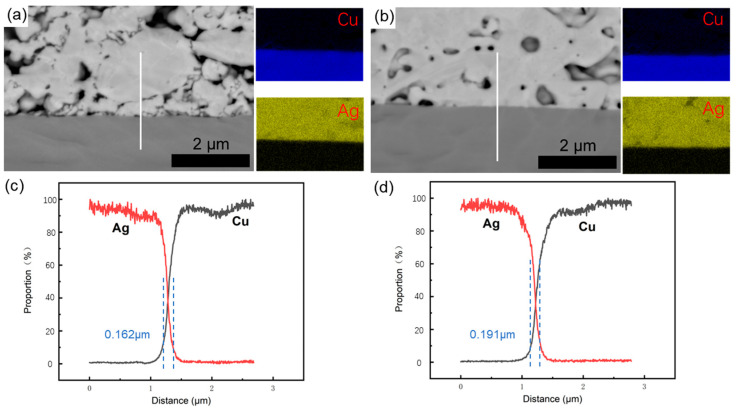
The EDS elemental profiles of (**a**) semi-sintered Ag joints and (**b**) sintered Ag joints. (**c**,**d**) show those at the lines indicated as white lines in (**a**,**b**).

**Figure 12:**
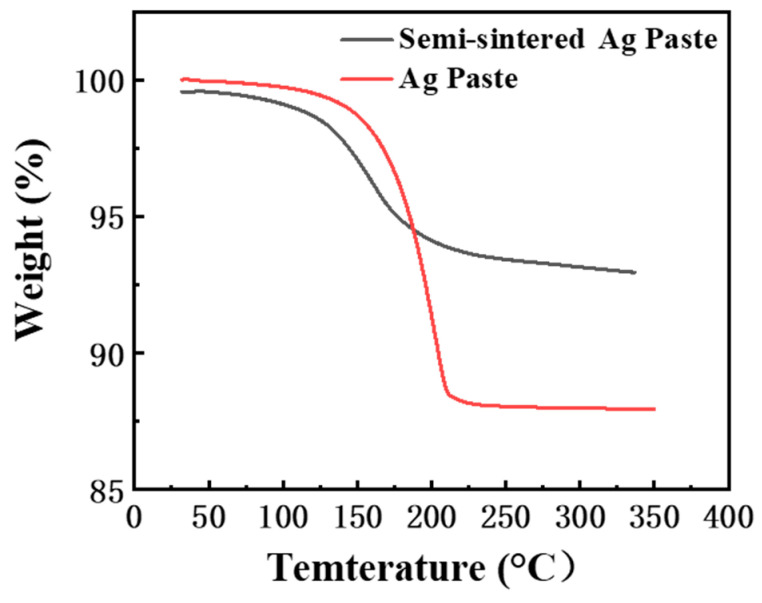
Thermogravimetric analysis of two pastes.

The porosity and interface connection rate of the sintered body are calculated by using the software ImageJ (Version: ImageJ-150-win-java8) to adjust the contrast, brightness, and threshold, and calculate the ratio of the pore area to the total area within a certain threshold range, as shown in Figure 10. The connection rate of the Ag-Cu interface of the sintered Ag joint is 73.25%, which is higher than the 63.58% of the semi-sintered Ag joint, indicating that the connection quality between the sintered Ag layer and Cu substrate is better. At the same time, the porosity of the sintered Ag body is lower than that of the semi-sintered Ag body. Through the energy spectrum analysis of the diffusion behavior of Cu and Ag elements at the interface (Figure 11), it is found that a certain degree of inter-diffusion has occurred at the interface of both samples. From the perspective of the vertical diffusion distance, the diffusion range of the Ag-Cu interface of the sintered Ag joint is slightly larger than that of the semi-sintered Ag joint, so its interface connection rate is higher. In summary, the differences in the interface connection rate, porosity, and inter-diffusion distance of the sintered Ag joint and the semi-sintered Ag joint at the microscopic level lead to the differences in their shear-tensile test results. Organic solvents such as diluents, binders, and dispersants are often contained in Ag paste. The residue of organic solvents during the sintering process of silver paste will hinder the densification of the sintering process [19,20,21,22]. By measuring the thermogravimetric analysis curves of the two Ag pastes (Figure 12), it is found that most of the organic substances in the Ag paste have been removed at 200 °C. At the sintering temperature of 300 °C, the weight of the Ag paste is reduced to 87% and has tended to be stable, while the semi-sintered Ag paste is reduced to 92% and is still continuously decreasing, indicating that more organic solvents are left in the semi-sintered Ag paste.

**Figure 13:**
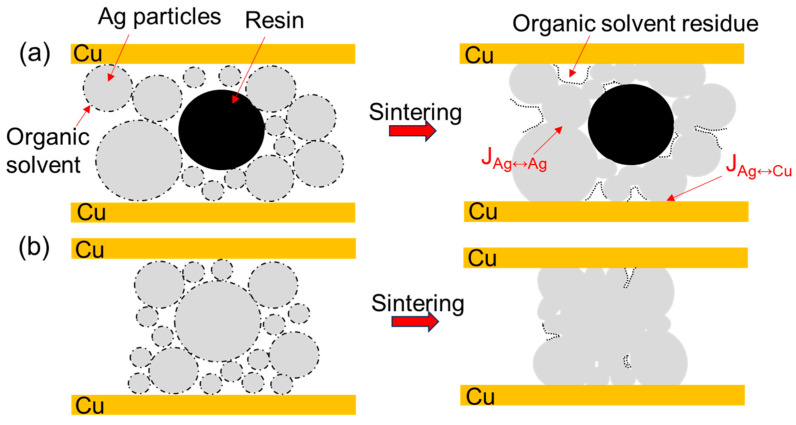
Schematic diagram of sintering densification process (**a**) semi-sintered Ag joints, and (**b**) sintered Ag joints.

The sintering densification processes of the two Ag pastes are shown in Figure 13. Before sintering, Ag particles are piled up with each other and wrapped in organic solvents. At the initial stage of sintering when the temperature is low, the volatilization and decomposition of organic solvents mainly occur, and the surface diffusion mechanism is dominant at this time. When the sintering temperature is increased and the organic solvents are further volatilized and reduced, the contact area between particles becomes larger, and the grain-boundary diffusion becomes the dominant mechanism. Meanwhile, nano-silver particles have both high surface energy and more grain boundaries. At this stage, the sintering necks are coarsened and grown, and the pores in the structure are closed to form a dense sintered body, that is, the sintered Ag body structure. Asoro et al. [23] proposed that some sintering necks will preferentially grow in places where the organic shells are thinner. Although the existence of organic solvents will not completely prevent the diffusion of particles, the sintering process will be significantly slowed down. Therefore, more organic substances left in the semi-sintered Ag paste will hinder the process of sintering densification. Moreover, the existence of resins will also hinder the contact between particles and the diffusion of Ag atoms. In addition, the proportion of nano-silver particles in the semi-sintered Ag paste is small, with the result that semi-sintered Ag paste still mainly has surface diffusion in the later stage of sintering, the degree of sintering densification is low, and some particles still maintain their original particle shapes after sintering. Therefore, the semi-sintered Ag paste sintering is mainly based on surface diffusion, while the Ag paste sintering is the result of the combined action of surface diffusion and grain-boundary diffusion. In the experiment on the influence of different metal-plating layers on the interface fracture toughness of sintered Ag by Wang et al. [24], it was proposed that the difference in the inter-diffusion behavior between Au, Cu, and the sintered Ag layer is the fundamental factor affecting the interface fracture behavior. In the experiment in this paper, the larger surface diffusion rate of the Ag paste can promote sufficient diffusion of Ag atoms on the surface of the Cu substrate, and increase the contact area between Ag atoms and Cu atoms, while at the same time, the diffusion activation energy of the grain boundaries is lower, which can promote the rapid inter-diffusion of Ag and Cu atoms through the grain boundaries, and form a tight connection between the sintered Ag layer and the Cu substrate, so the interface connection rate of the sintered Ag joint is higher.

It can be seen from the fracture surfaces of the tensile samples in Figure 8 and Figure 9 that the fracture modes of the sintered Ag joint and the semi-sintered Ag joint are the same, and both fractures occur along the Ag-Cu interface. The difference is that a large amount of sintered Ag remains on the fracture surface of the sintered Ag joint, and obvious ductile deformation can be observed. The silver remaining on the Cu surface at the semi-sintered fracture retains its original particle characteristics. Under the action of tensile stress, whether it involves the semi-sintered Ag joint or sintered Ag joint, the Ag-Cu interface is weaker than the sintered Ag layer and becomes the place where cracks originate and grow. Since the Ag-Cu interface is a heterogeneous interface and there is an incoherent interface in the Ag-Cu connection, the degree of ductile deformation is worse than that of silver itself, resulting in continuous stress concentration at the interface and becoming the place where cracks are formed [25]. The better interface connection quality of the sintered Ag joint contributes to better tensile strength. Keisuke Wakamoto et al. [26] compared the uniaxial tensile strengths of nano-sintered silver film and pure silver film and found that the fracture surface of the sintered silver film showed ductile deformation. Since the Ag-Cu connection area at the sintered Ag joint interface is higher than that of the semi-sintered, the more significant ductile deformation of the sintered Ag joint—more significant than that of the semi-sintered—requires further energy input, thus showing higher tensile strength. In the experiment where Keisuke Wakamoto et al. [27] compared the tensile properties of the sintered silver film at room temperature and 300 °C, the cracks in the sintered silver film at room temperature are generated at the pores and propagate along the grain boundaries, causing large-scale damage. Therefore, more pores at the semi-sintered Ag joint interface will also become the crack generation points, leading to the tensile failure of the joint.

It can be seen from the fracture surfaces of the shear tests in Figure 8 and Figure 9 that both are in the mode of composite fracture. Microscopically, on the one hand, the quality of the sintered Ag joint Ag-Cu interface is excellent; on the other hand, due to the high porosity of the semi-sintered silver layer and most of the pores being in the shape of acute angles, under the action of shear stress, stress concentration is likely to occur at the holes, and cracks are quickly generated and propagated in the silver layer. These factors lead to the lower shear quality of the semi-sintered Ag joint. In the shear tests of sintered silver at different temperatures by Yao and Gong [28] and Ma et al. [29], the porosity decreases with the increase in temperature, and the shear strength increases with the decrease of porosity. This also verifies the conclusion in this paper. Yang et al. [25] proposed that Young’s modulus of the sintered silver structure is mainly determined by the porosity. The increase of porosity leads to an increase in the load per unit area of the sintered structure and is more likely to cause the amplification and concentration of local stress. The porosity of the semi-sintered Ag structure is higher than that of the sintered Ag. When the shear sample is subjected to external force in the macro elastic deformation stage, the higher porosity causes more areas to enter the early plastic deformation. As the external stress load gradually increases, these stress concentrated areas will first exceed the stress limit of the material, resulting in local fracture. This stress concentration effect not only accelerates the failure process of the semi-sintered Ag joint but also significantly reduces the overall mechanical properties. This is consistent with the results of the calculation model by Zabihzadeh et al. [30]. Pores increase the average stress amplification factor and also increase the peak stress amplification factor, resulting in an increase in local stress near them. Although the existence of semi-sintered resin can hinder the crack path and prolong the crack path, under the action of shear stress, the heterogeneous interface where the resin and the Ag surface are not bonded is distorted and enlarged, but instead provides a path for the crack. When the shear force acts on the Ag layer, at this time, the Ag layer is subjected to two mutually perpendicular forces. When the interface in the middle area of the Ag-Cu interface is well bonded while the Ag layer itself is weaker, the force perpendicular to the Ag-Cu diffusion direction will cause the crack on the interface side to turn to the Ag layer.

**Figure 14:**
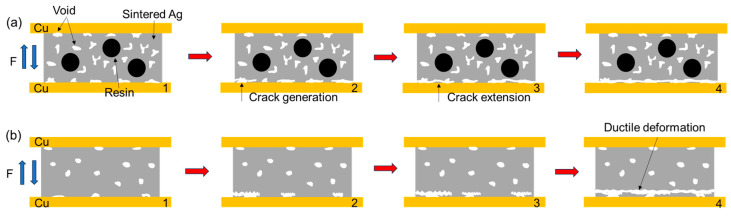
Schematic diagram of tensile fracture of two sintered joints (**a**) semi-sintered Ag joints; and (**b**) sintered Ag joints. From 1 to 4 is the fracture process of the sintered joint, 1 is the original organization.

**Figure 15:**
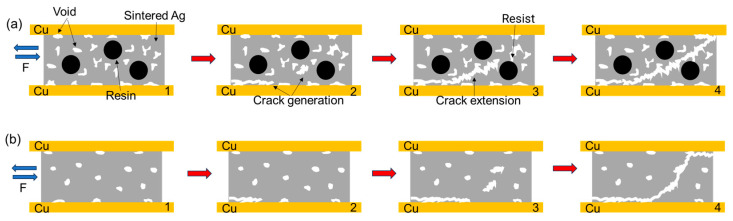
Schematic diagram of shear fracture of two sintered joints (**a**) semi-sintered Ag joints; and (**b**) sintered Ag joints. From 1 to 4 is the fracture process of the sintered joint, 1 is the original organization.

In summary, Figure 14 and Figure 15 show, respectively, the schematic diagrams of the tensile and shear fracture modes of the two sintered Ag joints. During the tensile process, the fracture occurs at the Ag-Cu interface. The semi-sintered Ag joint has a low interface connection rate and more pores. Stress concentration is likely to occur preferentially at the pores, leading to the generation of cracks (Figure 14(a2)), and then under the action of tensile stress, it quickly spreads to the entire Ag-Cu interface, resulting in failure (Figure 14(a4)). In the sintered Ag joint, the Ag-Cu interface has better connection quality, as the possibility of crack generation is smaller, certain ductile deformation is exhibited during the tensile process, and the fracture location is closer to the sintered silver layer at the interface (Figure 14(b4)). During the shear process, the pores at the Ag-Cu interface of the semi-sintered Ag joint, as weak parts, preferentially generate cracks, and the acute-angled holes in the sintered body generate stress concentration at the tips (Figure 15(a2)). Under the action of shear force, the cracks quickly expand along the holes. When encountering the resin, the resin can resist the expansion of the cracks, but the resin and the sintered Ag body also belong to a heterogeneous interface. Under the action of stress, the cracks then expand from the Ag body beside the resin (Figure 15(a3)). Finally, damage and failure are caused at both the Ag-Cu interface and the sintered Ag layer, showing a composite fracture mode (Figure 15(a4)). In the sintered Ag joint, on the one hand, the Ag-Cu interface has better connection quality; on the other hand, it has fewer holes and the shapes are more rounded, which is not conducive to the generation and expansion of cracks, and more energy is required when finally causing shear failure (Figure 15b), so the shear strength of the sintered Ag joint is higher.

## 4. Conclusions

In the present work, by comparing the shear and tensile fracture surfaces and microstructures of sintered Ag joints and semi-sintered Ag joints, the differences in the fracture mechanisms of the two types of Ag joints are revealed. The conclusions are as follows:

(1) In the tensile test, both joints are in Ag-Cu interface fracture mode, and in the shear test, composite fracture mode occurs.
(2) Due to the resins and organic substances blocking the diffusion of Ag atoms, the semi-sintered Ag joint is mainly surface diffusion-dominated during the sintering densification process, while the sintered Ag joint is the result of the combined action of surface diffusion and grain-boundary diffusion, resulting in a higher porosity and a lower interface connection rate in the semi-sintered Ag joint.
(3) During the tensile process, cracks are preferentially generated and propagated in the pores at the interface. The sintered Ag joint has a higher interface connection rate and better plasticity in the silver layer, so it has a high tensile strength.
(4) During the shear process, there are more pores in the sintered body and at the Ag-Cu interface of the semi-sintered Ag joint, causing stress concentration and rapid failure.

## Data Availability

The original contributions presented in this study are included in the article. Further inquiries can be directed to the corresponding authors.
